# Obituary

**Published:** 1877-11

**Authors:** 


Obituary.—The sad news of Dr. Paul F. Eve's sudden death, in Nashville, on the 3d of November, reached us first through the secular press. Truly, the medical profession mourns the loss of a leading venerable member, the Nestor of American surgery, and of world-renowned reputation.

So widespread is the fame of this laborious and distinguished surgeon, that any eulogy we might dictate would but repeat what is already in the minds and hearts of the public and the profession. As the recipient in early life of his instruction, however, we may feel more keenly the sad loss than those less intimate with his early history.

A special train was furnished to carry his remains to his old home in Augusta, Ga., where his dust will mingle with that of his friends and relatives who have gone before.

*Peace to his remains!*

ERRATA FOB OCTOBER No.—Page 393, 17th line from top, for *advantages* read *physicians;* page 396. for *botanist* read *botanic*.

